# Paraffin-Multilayer Graphene Composite for Thermal Management in Electronics

**DOI:** 10.3390/ma16062310

**Published:** 2023-03-13

**Authors:** Adriana Elena Balan, Ali AL-Sharea, Esmaeil Jalali Lavasani, Eugenia Tanasa, Sanda Voinea, Bogdan Dobrica, Ioan Stamatin

**Affiliations:** 1 Faculty of Physics, University of Bucharest, 3NanoSAE Research Center, 077125 Bucharest-Măgurele, Romania; 2 Iraqi Atomic Energy Commission–Baghdad, Baghdad P.O. Box 765, Iraq; 3 Department of Physics, Politehnica University of Bucharest, 313 Splaiul Independentei, 060042 Bucharest, Romania

**Keywords:** phase change materials, paraffin-multilayer graphene, latent heat, heat sink

## Abstract

Multilayer graphene–paraffin composites with different contents of graphene (0–10 wt.%) were prepared using an ultra-high shear mixer. The aim is to improve the heat transfer in paraffin wax, which will lead to more-efficient thermal buffering in electronic applications. The multi-layer graphenes obtained by supercritical fluid exfoliation of graphite in alcohol were investigated by Raman spectroscopy, scanning electron microscopy and atomic force microscopy. Interesting morphological features were found to be related to the intercalation of paraffins between the multilayer graphene flakes. Thermal properties were also investigated in terms of phase change transition temperatures, latent heat by differential scanning calorimetry and thermal conductivity. It was found that the addition of graphene resulted in a slight decrease in energy storage capacity but a 150% improvement in thermal conductivity at the highest graphene loading level. This phase-change material is then used as a thermal heat sink for an embedded electronic processor. The temperature of the processor during the execution of a pre-defined programme was used as a performance indicator. The use of materials with multilayer graphene contents of more than 5 wt.% was found to reduce the processor operating temperature by up to 20%. This indicates that the use of such composite materials can significantly improve the performance of processors.

## 1. Introduction

In response to environmental and climate change issues, the scientific community has been at the forefront of the search for the most sustainable technological solutions to energy problems. In addition to developing new solutions for generating electricity with low emissions, progress has also been made in developing ways to store energy. In this context, the management of thermal energy is an important piece of the puzzle. One challenging approach is to implement thermal energy management solutions using passive phase-change materials (PCMs) [1,2,3]. The ability to store and release large amounts of energy during phase change processes, i.e., latent heat, is the key feature of PCMs. PCMs are already being used for thermal energy storage in building materials, refrigeration systems, heat pumps, electronic devices, aerospace applications, etc. [4,5,6,7,8]. In electronic devices, superior computing performance and miniaturisation have sharply increased the density of thermal energy generation, requiring efficient management of heat dissipation.

For efficient cooling, a PCM should ideally have high latent heat, high specific heat, good thermal conductivity, chemical stability, non-flammability, and a low manufacturing cost [9]. Most organic and inorganic PCMs have latent heat in the interval 150–250 kJ/kg with a thermal conductivity of less than 0.5 W/mK [10]. N-alkanes and their mixtures, known as paraffin waxes, are the most commonly used organic PCMs. Pure n-alkanes are linear chains of methylene groups ending with two methyls: CH_3_-(CH_2_)_n-2_ -CH_3_. They have a latent heat of 244 kJ/kg (n = 18, octadecane) up to a maximum of 285 kJ/kg (n = 100, hectane) in the temperature interval 28–115 °C [11]. Combinations of n-alkanes with small amounts of iso- and cycloalkane and some additives are available under various trademarks. They cover the latent heat interval of 150–250 kJ/kg with a density of 800–920 kg/m^3^ and melting temperatures from RT to 100 °C and are non-toxic, non-corrosive, and low-cost. However, the low thermal conductivity is a major obstacle to their widespread use.

One possible approach is the use of various structures such as fins, metal foam and metal inserts to promote heat transfer [12,13]. In some applications, this is an easy and cheap solution, but it has the disadvantage that any metallic structure will add to the overall weight and volume of the system. Another solution is to use various additives, i.e., nanometric structures in a small volume fraction, to increase the actual thermal conductivity of the PCM [14]. The thermal transfer properties of PCMs can be improved by adding different materials with high thermal conductivity, such as nano-graphite sheets [15,16], metal foam [17], alumina, TiO_2_ [18], carbon fibre [19], carbon multiwall nanotubes [20] or expanded graphite [21,22,23,24,25]. Cong et al. [26] published a comprehensive review on the thickening and gelling agents as additives, aiming to improve the stability and stabilise the shape of thermal energy storage materials. Other studies have proposed more complex solutions that address not only the thermal conductivity issue but also the leakage behaviour, using self-assembled hybrid aerogel filled with carbon nanotubes in combination with reduced graphene oxide nanosheets [27]. Due to their high surface area, carbon nanostructures are used with various PCMs as additives or support structures through various interactions such as surface tension, capillary, van der Waals or hydrogen bonding [28]. Cheng et al. [29] proposed a shape-stabilised phase-change composite based on a 3D reduced graphene oxide decorated melamine sponge as a support and paraffin wax as a filler. The high thermal conductivity makes expanded graphite an ideal additive or support material for PCMs. Some studies focused on the effect of an expanded graphite particle size in paraffin composites [30,31]; others focused on the distribution and interaction between paraffin and expanded graphite during thermal cycling [32]. The melting and solidification processes have also been studied for paraffin–graphene aerogel composites, where the numerical simulation has been carried out using the volume average method [33].

Recent work has focused on understanding how graphene affects the effective thermal conductivity. Graphene, an sp^2^-bonded monolayer 2D carbon sheet with a hexagonal honeycomb lattice, has received considerable attention over the past few years for its numerous extraordinary properties. These include a high theoretical specific surface area (2630 m^2^ g^−1^) and excellent thermal conductivity (3000–5000 W m^−1^ K^−1^) [34]. The behaviour of paraffin molecules in the presence of graphene nanoparticles was studied by Babaei et al. [35] using molecular dynamics simulations. The results showed that there is a strong dependence of the thermal conductivity on the relative orientation of the PCM and the nano-inclusions. It appears that the graphene-based nanoparticles interact with the PCM molecules in such a way as to influence their intrinsic alignment. Directed ordering of the PCM molecules in close proximity to the graphene molecules is reported.

In this context, this paper presents an in-depth study of the thermal behaviour of multilayer graphene–paraffin composites. We propose a reliable fabrication method: (I) obtaining the multilayer graphenes by exfoliation of graphite in supercritical fluids using an alcohol-based solvent, followed by (II) their incorporation into a paraffin matrix using an ultra-high-shear mixer. The novelty of the study derives from the environmentally friendly method in terms of the chemicals used to obtain the graphene, followed by their incorporation through a simple mixing process. Another aspect is the simple method of rapid evaluation of heat sink performance by analysing data provided by the processor. The aim is to improve the thermal transfer in paraffin wax, leading to more efficient thermal buffering in electronic applications. Structural and morphological analyses as well as thermal properties have been studied. Subsequently, composite phase change material has been tested as a heat sink on an integrated electronic circuit. The temperature of the processor was monitored during the execution of a predefined programme as a performance indicator.

## 2. Materials and Methods

### 2.1. Materials

Pristine natural graphite (99%+ purity) was purchased from Asbury Carbons Inc. The PCM matrix and paraffin wax mp 58–62 °C were supplied by Sigma Aldrich. Other reagents: ethanol (99.5% Sigma-Aldrich, Saint Louis, Missouri, U.S.A.), sulfuric acid (Merck Inc., Darmstadt, Germany), and nitric acid (Sigma-Aldrich, Saint Louis, Missouri, U.S.A.), were used as delivered.

### 2.2. Preparation of Paraffin-Multilayer Graphene Composite

**Figure 1:**
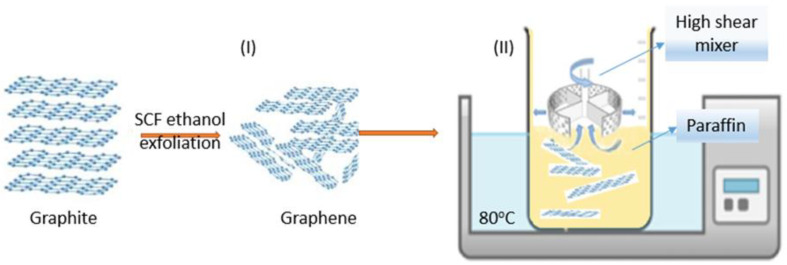
Multilayer graphene–paraffin composite preparation steps: (**I**) multilayer graphene was prepared by supercritical fluid exfoliation in ethanol aqueous solution and (**II**) multilayer graphene dispersion in paraffin matrix by high-shear mixer at 80 °C and 5000 rpm for 30 min.

The first step is graphene exfoliation in supercritical fluids (SCF) using an aqueous solution of ethanol. Previous studies have shown that the exfoliation process occurs when the energy required is compensated by the solvent –graphene interaction [36,37]. It requires solvents with surface energies close to that of graphene [38]. The graphite–solvent mixture was prepared as follows: 1 g of natural graphite was dispersed in 300 mL aqueous solution of ethanol:water = 4:1 molar ratio in an ultrasound bath for 10 min. The solution was placed in a stainless steel SCF reactor at 320 °C and 150 bar for 1 h while stirring (step I in Figure 1). The exfoliated graphite was filtered and washed several times with a high-purity solvent. Multilayer graphene was collected and vacuum dried for 24 h at 100 °C. Due to the strong Van der Waals force and great specific surface area, multilayer graphene flakes tend to agglomerate. On the other hand, paraffin wax has a very low surface tension of approximately 30 mN/m at 20 °C, decreasing with temperature, and high hydrophobicity [39]. In order to obtain a stable and uniformly dispersed PCM composite, an ultrahigh shear rate mixer (L5M-A, Silverson Machines, Inc., East Longmeadow, MA, U.S.A.) was used at 500 rpm for 5 min, followed by 5000 rpm for 30 min, at 80 °C (step II in Figure 1). Samples were then moulded as needed and left to crystalise at room temperature.

### 2.3. Characterisation Methods

Raman spectroscopy was performed for identifying specific features of graphenes, i.e., G and 2D bands, with Jasco NRS 3100 Raman (Japan), at 532 nm with a power of approximately 3 mW. To investigate the effect of multilayer graphene dispersion in a paraffin matrix, morphological characterisation was performed using scanning electron microscopy (SEM) using a JIB-4600F system (Tokyo, Japan). Before imaging, samples were fractured after liquid nitrogen immersion and the surfaces were coated with 3 nm platinum. Atomic force microscopy (AFM) topography images were obtained using an SPM-NTegra Prima AFM (NT-MDT, Russia), operated in semicontact mode, at a scanning rate of 1 Hz, using NSG01 probe series, with a 87–230 kHz resonant frequency and 1.45–15.1 N/m force constant. Samples were fractured after liquid nitrogen immersion and scanned in air at room temperature and with an approximately 40% relative humidity.

Thermal analysis was performed using a differential scanning calorimeter (DSC Mettler Toledo, model Star1, Switzerland) in a nitrogen atmosphere at a heating rate of 10 °C/min in the temperature range 20–90 °C. The 5–10 mg samples were sealed in aluminium pans. DSC curves were analysed with the DSC Standard Data Analysis Program associated with model Star1. Indium was used as a calibration reference of DSC. Each sample was treated for 30 min at 90 °C to lose the thermal memory effect.

Thermal conductivity was determined by the transient hot bridge method using a THB 100 with a THB/Sensor/B, produced by Linseis Messgeräte GmbH, Germany. The composite phase change material was tested as a passive thermal buffer on a Raspberry Pi 3 Model B+ integrated processor. PCM samples were placed in a copper crucible and placed on top of the processor using thermoconductive paste. The processor temperature while running a predefined program was monitored for all samples under the same experimental conditions.

## 3. Results

### 3.1. Raman Spectroscopy and Morphology Tests

**Figure 2:**
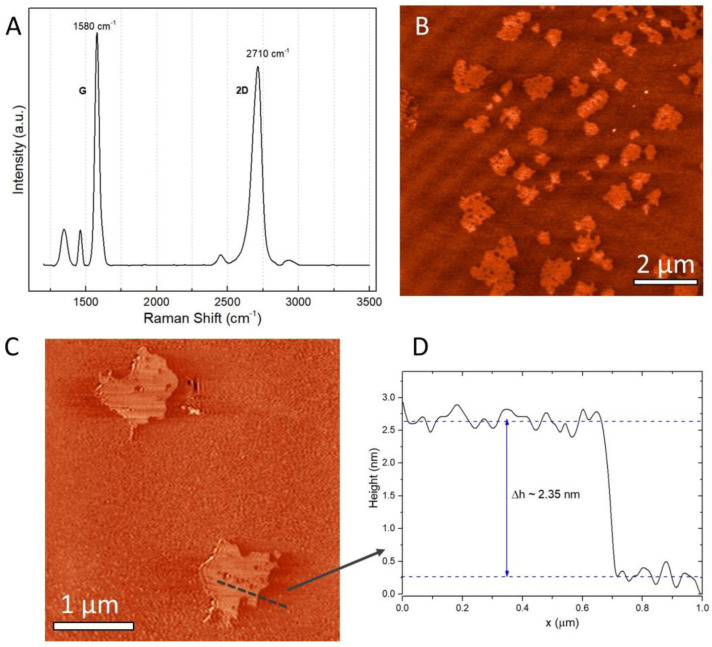
(**A**) Raman spectra of multilayer graphene obtained by supercritical fluid exfoliation; (**B**) AFM topography image of graphenes deposited on silicon wafer substrate, scan area 10 µm × 10 µm and (**C**) scan area 3.7 µm × 3.7 µm; (**D**) cross-section profile evidencing a multilayer graphene thickness of approximately 2.35 µm.

Raman spectroscopy on the multilayer graphene obtained by supercritical fluid exfoliation presented in Figure 2A shows the main features of graphene and graphite, the G band (~1580 cm^−1^) and 2D band (~2700 cm^−1^). Typical D, G and 2D peaks are present in all graphite exfoliation samples. The G band is a degenerate double phonon mode (E_2g_ symmetry) in the centre of the Brillouin zone, which originates from the in-plane vibration of *sp^2^* carbon atoms. The 2D band originates from a double-resonance Raman process. It is therefore closely related to the band structure of graphene layers, whereas that of graphite has a single peak. The G band is associated with double degenerate phonon modes in the Brillouin region of the Raman spectrum [40]. The shape of the 2D peaks and their intensity relative to the G peak can indicate the number of graphene layers. A very sharp, symmetrical 2D Lorentzian peak with twice the intensity of the G peak is characteristic of single-layer graphene [41]. As the number of layers increases, the 2D peak becomes broader, less symmetric and less intense, as shown in Figure 2A. From the shape and intensity of the 2D peak, it can be concluded that the graphene under investigation is a multilayer structure. Due to the method of obtaining the graphene, the D band remains at a low intensity.

**Figure 3:**
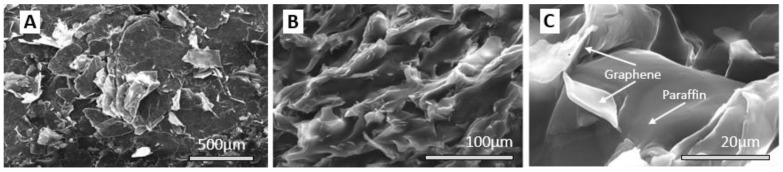
Microstructure of multilayer graphene (**A**,**B**) and 1% graphene–paraffin composite (PCM-1G) (**C**). Images were taken on sample cross-sections obtained by fracture of solid samples after liquid nitrogen immersion and then coated with 3 nm platinum.

The sample morphology is shown in Figure 2B,C, where AFM images for multilayer graphene obtained by exfoliation in supercritical fluids are presented. Samples were prepared by drop-casting graphene dispersion in water on a freshly cleaved mica substrate followed by air drying. The cross-section presented in Figure 2D shows a thickness of approximately 2.35 nm, which, according to other studies, corresponds to less than 10 layers of graphene [42,43,44]. The multilayer graphene flakes obtained by the synthesis in supercritical fluids have a wide dispersion in terms of lateral size, ranging in the interval 0.5 to 10 µm, as confirmed by the SEM images presented in Figure 3A,B.

In order to highlight the morphology of the sample, the surface under investigation was obtained by fracturing the sample after cooling in liquid nitrogen. Figure 3C shows, at different magnifications, the intercalation and homogeneous distribution of graphenes in the paraffin matrix.

**Figure 4:**
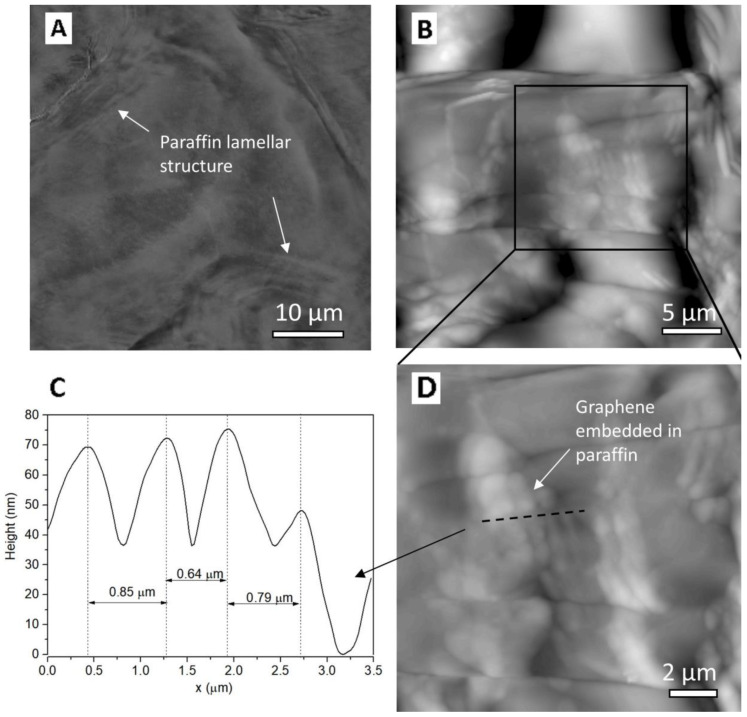
AFM images: (**A**) topography on paraffin sample, scan area 50 µm × 50 µm, (**B**) topography on 1% graphene–paraffin composite (PCM-1G) sample, scan area 25 µm × 25 µm; (**C**) topography on 1% graphene–paraffin composite (PCM-1G) sample, scan area 10 µm × 10 µm, shows carbon structures embedded in paraffin matrix; (**D**) cross-section profile evidencing an interlayer spacing of approximately 0.7 µm. The investigated surface was obtained by fracturing the liquid nitrogen frozen samples.

AFM topography images reveal a typical morphology of paraffin, i.e., lamellar structure (Figure 4A). The lamellar structure of paraffin derives from the crystallisation processes described by the conventional homogeneous nucleation and growth mechanisms occurring in the liquid phase [45]. Previous studies have reported that each lamella within the paraffin structure is about 7.6 ± 0.2 nm thick, corresponding to a molecular bilayer [46]. In the 2D topography images (Figure 4B,D), the distribution of graphene flakes in the polymer matrix can be observed, forming domains ranging in size from 0.5 to 10 µm, in agreement with SEM images. The formation of a paraffin–graphene interface is mostly driven by non-covalent interactions, and non-covalent bonds would be formed between paraffin and graphene, even in the presence of a vacancy in graphene [47]. One can note the cross-section profile evidencing an interlayer spacing of approximately 0.7 µm (see Figure 4C).

### 3.2. Thermal Analysis

**Figure 5:**
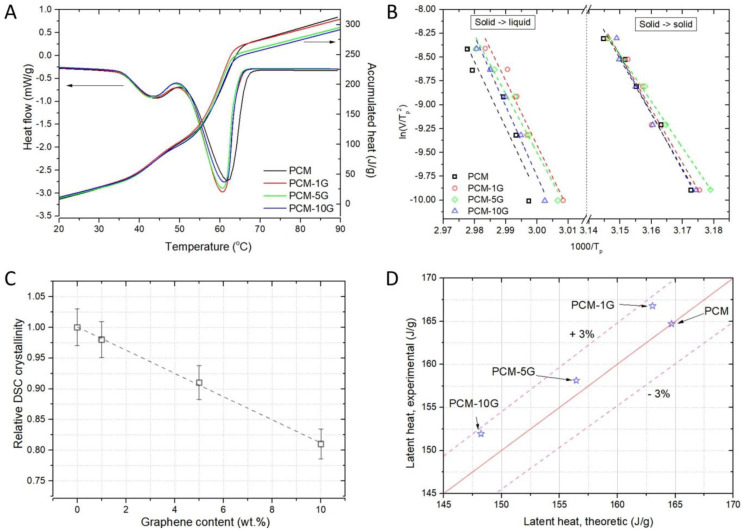
DSC analysis of paraffin- (PCM) and graphene-based composites: (**A**) heat flow curves vs. temperature and the corresponding accumulated heat recorded under 80 mL min^−1^ nitrogen flow, at a heating rate of 10 °C min^−1^; (**B**) graphic representation $ln\left(T_{p}^{2}/v\right)$ vs. $1/T_{p}$, where the slope of the line resulting from linear fitting is proportional to the activation energy according to the Kissinger equation; (**C**) relative DSC crystallinity dependence on the graphene content; (**D**) latent heat evaluated by integrating the corresponding peak on the DSC curves in (**A**) compared to the theoretically estimated values.

The paraffin and composite samples with graphenes obtained by synthesis in supercritical fluids at different concentrations (1, 5, 10 wt.%) have been investigated in terms of thermal behaviour. The DSC melting and crystallisation curves obtained at a heating/cooling rate of 10 °C/min are shown in Figure 5. Two first-order phase transitions are thus identified: (a) solid–solid transition at lower temperatures (40–50 °C) due to the transition from monoclinic to hexagonal pseudocrystal structure and (b) solid–liquid phase transition at higher temperatures (50–70 °C) and isotropisation. Table 1 shows the data extracted from the DSC curves, namely: melting (*t_m_*) and crystallisation (*t_c_*) temperature along with the activation energies calculated by the Kissinger model as described below. Melting and crystallisation temperatures do not change dramatically with the addition of graphene, in agreement with other studies [1,48,49]. However, the activation energy and latent heat do change, as expected.

The activation energy of the thermodynamic processes, i.e., solid–solid and solid–liquid transitions, respectively, was estimated using the Kissinger equation [50]:

(1) $$\frac{E_{a}v}{kT_{p}^{2}}=Ae^{\frac{E_{a}}{kT_{p}}}$$

where $E_{a}\;\left(\frac{kJ}{mol}\right)$ is the activation energy of the proccess, v is the heating rate, k is the Boltzmann constant, $T_{p}$ is the transition temperature, and A is the pre-exponential factor. Using the graphic representation $ln\left(T_{p}^{2}/v\right)$ vs. $1/T_{p}$, the activation energy is determined from the slope of the line resulting from linear fitting. In our study, five different heating rates were used: 5, 10, 15, 20, and 25 °C/min (Figure 5B). The activation energy generally does not change dramatically with the concentration of carbonic material in the paraffin matrix, as shown in Table 1. The exception is the sample with the highest additive concentration, wherein the activation energies are lower for both processes. The interaction between carbon and paraffin at the interface between the two materials is most likely weaker in this case. This interaction becomes less dominant once a percolating threshold is exceeded. The movement of clusters of molecules is the most likely mechanism for mass transfer at the crystal-melt interface during the melting process [51]. In turn, it is well known that the activation energy of diffusion is proportional to the size of the diffusing species. The activation energy of diffusion in the interfacial region can be expected to be greater as long as such clusters are larger than the molecules or their aggregates present in the regular melt [52].

The disorder effects generated by the multilayer graphene significantly affect the crystallisation properties of the composites. The internal interactions between the paraffin molecular chains are slightly inhibited, hindering the nucleation process and crystal network growth, as the multilayer graphene content increases. This leads to lower enthalpy of crystallisation and a reduction in the DSC crystallinity of the paraffin matrix. To obtain a more intuitive picture of the additive influence on matrix crystallinity, instead of estimating a degree of crystallinity, defined as the ratio between melting enthalpy and melting heat if the polymer was 100% crystalline, a relative DSC crystallinity, $X_{c,rel}$, is calculated using the following relationship [53]:

(2) $$X_{c,rel}=\frac{X_{c,PCM-fG}}{X_{c,PCM}}=\frac{\Delta H_{m,\;PCM-fG}}{\Delta H_{m,\;PCM}}\left(1-f_{G}\right)$$

where $X_{c,PCM-fG}$ and $X_{c,PCM}\;$ are the degree of crystallinity of a paraffin multilayer graphene compound and pure paraffin, $\Delta H_{m,\;PCM-fG}$ and $\Delta H_{m,\;PCM}$ are fusion enthalpies determined as the area of the melting effect considering a linear baseline and $f_{G}$ is the weight fraction of multilayer graphene in the paraffin matrix. Since we address the structural and functional modifications of the additive to the PCM material, it is more convenient to refer to a relative quantity. Figure 5C indicates the linear dependence of the relative DSC crystallinity on the graphene content. It appears that multilayer graphenes interact with paraffin polymeric chains affecting their basic alignment and, therefore, the natural dynamics.

Moreover, the specific heat of the paraffin–graphene mixture should be lower than that of paraffin. This is also confirmed by the obtained results, except at higher temperatures, where it can be seen in Figure 5A that for concentrations lower than 5%, the specific heat has higher values. The atypical increase could be caused by an additional energy storage mechanism due to interactions at the high surface area graphene/paraffin interface. Another aspect is the decrease in intermolecular space in paraffin due to its adhesion to the surface of the carbon layers.

Latent heat was also evaluated by integrating the corresponding peak on the DSC curves in Figure 5A and graphically represented by comparison with the theoretically estimated values (see Figure 5D) using the following formula:

(3) $$\Lambda_{PCM-xG}=\Lambda_{PCM}\cdot \left(1-f_{G}\right)$$

where $\Lambda_{PCM-xG}$ is the latent heat of the composite PCM material, $\Lambda_{PCM}$ the PCM latent heat determined experimentally, and $f_{G}$ is the graphene weight fraction.

It is evident that the latent heat decreases with the mass fraction of the additive. The experimental values confirm this within the 3% range. The local agglomeration that may occur in the composite mixture and the overall molecular potential energy of the material also affect the latent heat.

### 3.3. Thermal Conductivity and Heat Transfer Evaluation

**Figure 6:**
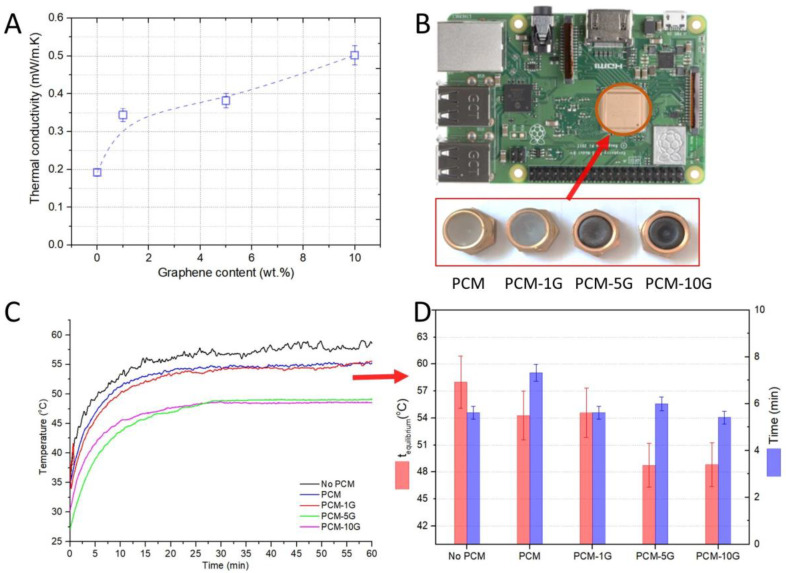
(**A**) Thermal conductivity of the composite samples with different graphene concentrations. (**B**) The Raspberry Pi 3 model B+ motherboard and PCM samples used for thermal buffering. (**C**) The internal temperature of the processor evolution in time shows a significant decrease of up to 10 °C as the graphene content increases. (**D**) The equilibrium temperature and the time required to reach the stable temperature.

The heat transfer rate during phase transitions is significantly inhibited and the charging and discharging time is extended due to the poor thermal conductivity of PCMs. A significant improvement in thermal conductivity can be achieved by adding graphene to paraffin. The thermal conductivities of the composite PCMs with the different mass fractions of the multilayer graphene are shown in Figure 6A. As expected, the thermal conductivities of composite PCMs show an upward trend with an increase in the mass fraction of multilayer graphene. For example, the relative enhancement in thermal conductivity of the 10 wt.% graphene composite is 150%. The thermal conductivity shows a strong dependence on the relative orientation of the PCM and nanoinclusions, which is also related to the crystallinity, as suggested by other studies [54]. Graphenes interact with the molecular chains of the PCM, disrupting their fundamental orientation, as shown also in the SEM images in Figure 4.

Heat transfer tests were performed using a Raspberry Pi 3 model B+ motherboard. The motherboard was programmed to run a computational program so that the processor was loaded as close as possible to its maximum operating power, while also recording the processor temperature evolution over time. PCM samples were placed on top of the processor, as shown in Figure 6B, for studying their impact on the operating temperature of the processor. Thus, the interpretation of the temperature-vs.-time graph obtained under similar load conditions, in the same time interval, allows the comparative evaluation of the samples (Figure 6C). The results show that the paraffin–graphene composite material with a 10 wt.% graphene concentration decreases the operating temperature by 10 °C, with a slower time evolution of the equilibrium temperature by about 3 min (Figure 6D). Temperature fluctuations are also attenuated, resulting in better processor operation.

## 4. Conclusions

Multilayer graphene–paraffin composite PCMs with different mass fractions were prepared, and their thermal behaviour was extensively investigated. The following conclusions can be drawn from the above research:

(1) The morphology of the PCM composites, both SEM and AFM images, showed a uniform distribution of the multilayer graphene within the paraffin matrix. Paraffin molecular chains form interlayers of approximately 0.7 µm between the graphene flakes, demonstrating an intimate contact that promotes thermal contact.

(2) The phase change temperatures, melting and crystallisation are only slightly changed by the addition of multilayer graphene compared with pure paraffin. With regard to the latent heat, the experimental results follow the theoretical estimation of the impact of the graphene weight percent in the PCM.

(3) As expected, the thermal conductivities of composite PCMs show an upward trend with an increasing mass fraction of multilayer graphene. For example, 10 wt.% graphene in the PCM composite exhibited a 150% relative increase in thermal conductivity.

(4) We have proposed an original experimental setup for testing the thermal buffering behaviour. Samples were placed in special crucibles directly on top of the processor of a Raspberry Pi 3 model B+ motherboard. A computational program was then run to load the processor as close as possible to its maximum operating power, and the internal temperature vs. time data was plotted under similar processor load conditions, in the same time interval. The results show that a 10 wt.% graphene load reduces the operating temperature by 10 °C, with a slower time evolution of the equilibrium temperature by about 3 min. Another important aspect is that temperature fluctuations are attenuated, an important factor in processor performance.

## Figures and Tables

**Table 1:** Melting and crystallisation temperatures and activation energies for the solid–solid and solid–liquid phase transitions estimated using the Kissinger model.

			Solid–Solid			Solid–Liquid		
	t_m_ (°C)	t_c_ (°C)	E_a_ (kJ/mol)	ln A	R	Ea (kJ/mol)	ln A	R
PCM	61.19	53.64	480.42	184.46	0.978	574.20	208.39	0.849
PCM-SCF-1%	60.54	54.36	469.73	180.42	0.962	560.52	203.94	0.955
PCM-SCF-5%	60.47	53.46	498.62	191.42	0.975	610.36	221.68	0.980
PCM-SCF-10%	60.76	53.70	403.79	155.29	0.996	511.11	185.94	0.962

## Data Availability

The data presented in this study are available on request from the corresponding authors.
